# Comparative Analysis of Biological Sphingolipids with Glycerophospholipids and Diacylglycerol by LC-MS/MS

**DOI:** 10.3390/metabo4010098

**Published:** 2014-01-27

**Authors:** Hideo Ogiso, Makoto Taniguchi, Shinichi Araya, Shinya Aoki, Lusi Oka Wardhani, Yuka Yamashita, Yoshibumi Ueda, Toshiro Okazaki

**Affiliations:** 1 Department of Hematology/Immunity, Kanazawa Medical University, Uchinada-machi, Kahoku-gun, Ishikawa 920-0293, Japan; E-Mails: hogiso@kanazawa-med.ac.jp (H.O.); araya-s@kanazawa-med.ac.jp (S.Ar.); aokis@kanazawa-med.ac.jp (S.Ao.); yama73@kanazawa-med.ac.jp (Y.Y.); yueda@kanazawa-med.ac.jp (Y.U.); 2 Medical Research Institute, Kanazawa Medical University, Uchinada-machi, Kahoku-gun, Ishikawa 920-0293, Japan; E-Mail: matanigu@kanazawa-med.ac.jp; 3 Division of Molecular Pathology, Faculty of Medicine, Tottori University, Nishi-cho, Yonago 683-8503, Japan; E-Mail: lusioka@gmail.com

**Keywords:** sphingolipid, glycerophospholipid, diacylglycerol, sphingomyelin, ceramide, sphingosine-1-phosphate, phosphatidylcholine, liquid chromatography, mass spectrometry, lipidomics

## Abstract

Liquid chromatography-electrospray ionization mass spectrometry (LC-MS) is an effective and popular technique used in lipid metabolomic studies. Although many LC-MS methods enabling the determination of sphingolipid molecular species have been reported, they do not cover a broad range of sphingolipid metabolites with expanding glycerophospholipids (GPLs) and diacylglycerol (DAG). In this study, we developed an approach for the comprehensive analysis of sphingolipids, GPLs and DAG molecular species in a biological sample, without alkaline hydrolysis. After validating the reliability of this approach, we analyzed tissue lipids of sphingomyelin synthase 2-knockout mice and found that changes in sphingolipid metabolism in the liver affect the level of docosahexaenoic acid-containing GPLs. Our method analyzes GPLs and DAG, as well as sphingolipids within biological samples and, thus, will facilitate more comprehensive studies of sphingolipid metabolism in pathology and diagnostics.

## 1. Introduction

Sphingolipids, which have a characteristic sphingoid base structure, include several classes of molecules, such as sphingosine (Sph), sphingosine-1-phosphate (S1P), ceramide (Cer), glucosylceramide (GlcCer), sphingomyelin (SM) and ganglioside [1]. These lipids are interconvertible in biological systems. For instance, S1P and Cer have counter-regulatory functions in cell proliferation and apoptosis, respectively [2,3,4,5]. Therefore, the relative levels of sphingolipids and their metabolites are important in determining cell fate (*i.e*., life or death). In fact, sphingolipids are associated with several diseases, such as cancer, obesity and atherosclerosis [6,7,8,9,10,11,12,13]. In this context, several mass spectrometry (MS) methods for comprehensive sphingolipid analysis have been reported [14,15,16,17,18,19,20,21]. The current analytical procedure for sphingolipids includes an alkaline hydrolysis treatment to cleave glycerolipids, to enable the determination of SM [15,17,18]. Therefore, this sample pretreatment method, including alkaline hydrolysis, does not cover a broad range of other glycerolipids, such as phosphatidylcholine (PtdCho) and diacylglycerol (DAG), which are involved in SM synthesis. In addition, concerns have been raised about potential artificial changes caused by the alkaline treatment during the sample preparation [17]. On the other hand, large amounts of coexisting basic lipids, such as PtdCho and phosphatidylethanolamine (PtdEtn), may impede the detection of the acidic components, even when the lipid species are separated by reversed phase LC [22,23]. In the present study, we developed an LC-MS/MS method without alkaline hydrolysis, for the comprehensive determination of Sph, S1P, Cer, ceramide-1-phosphate (Cer1P), monohexosylceramide (HexCer), monosialoganglioside (GM3), SM, PtdCho, PtdEtn, phosphatidylserine (PtdSer), phosphatidic acid (PtdOH), phosphatidylinositol (PtdIns), phosphatidylglycerol (PtdGro) and DAG with different fatty acid compositions. First, we designed protocols for the limited extraction of lipids ranging from S1P to DAG, but excluding triacylglycerol (TAG), which is too hydrophobic to assess together with S1P. Next, we employed diethylaminoethyl (DEAE)-cellulose chromatography to separate the acidic lipids from the large amounts of basic and neutral lipids present in biological samples [22,23]. These extracts were then analyzed by LC-MS/MS, to determine the molecular species level of each lipid class with minimization of the ion-suppression that may occur when a lipid extract from a complex biological sample is subjected to an electrospray ionization MS analysis. Using this approach, we examined the lipid composition of tissues from SM synthase 2-knockout (SMS2-KO) mice. Our results indicated that this method would be useful for comprehensive studies of sphingolipid metabolism in cellular responses and pathology.

## 2. Results and Discussion

### 2.1. Extraction of Sphingolipids, GPLs and DAG

**Figure 1:**
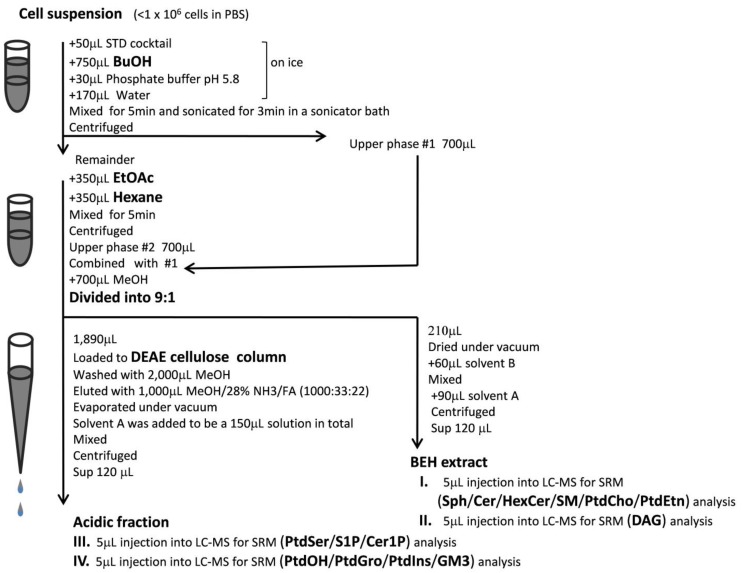
Flow chart illustrating sphingolipid, glycerophospholipid (GPL) and diacylglycerol (DAG) sample preparation and analysis. Butanol and ethyl acetate/hexane (1:1) were used for the first and second extractions, respectively. To reduce the ion suppression effects associated with large amounts of phosphatidylcholine (PtdCho) and phosphatidylethanolamine (PtdEtn) in the sample, a DEAE fractionation step was introduced for the preparation of acidic lipids. Depending on the analyte assayed, four different MS methods (I–IV) were used for higher sensitivity. Further details are available in the Materials and Methods Section. SRM, selected reaction monitoring; Sph, sphingosine; Cer, ceramide; HexCer, monohexosylceramide; SM, sphingomyelin; PtdSer, phosphatidylserine; S1P, sphingosine-1-phosphate; Cer1P, ceramide-1-phosphate; PtdOH, phosphatidic acid; PtdGro, phosphatidylglycerol; PtdIns, phosphatidylinositol; GM3, monosialoganglioside; STD, standard solution; FA, formic acid.

**Figure 2:**
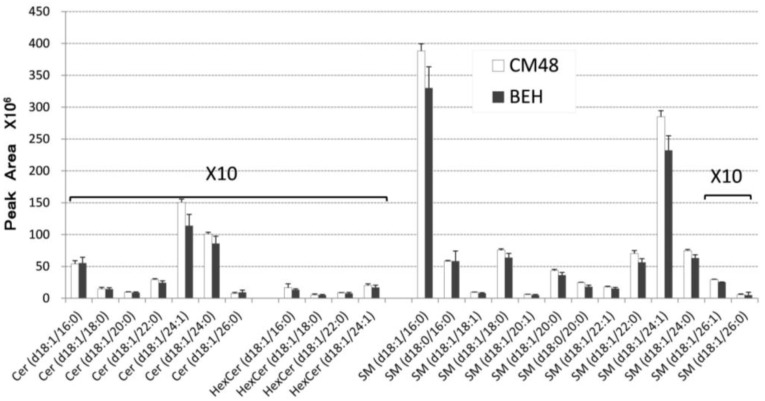
Comparison of the recovery of sphingolipids extracted by the chloroform/methanol at 48 °C (CM48) and butanol/ethyl acetate/hexane (BEH) methods. Lipids from 1 × 10^6^ WR19/Fas-SMS1 (WR/SMS1) [24] cells were extracted using either the CM48 or BEH method (see text). Both extracts were analyzed by LC-MS/MS. Values are expressed as the mean ± SD (*n* = 3).

Sphingolipid metabolism is linked to GPL metabolism via PtdCho and DAG, which are involved in an interchange reaction between Cer and SM. Membrane microdomains that are enriched in sphingolipids are thought to play an important role in membrane receptor function [24,25,26]; therefore, analyzing the relative levels of the various sphingolipids and GPLs is critical for understanding biological systems. In this study, we developed a comprehensive analytical method to determine the levels of individual species of sphingolipids, GPLs and DAG. First, we designed protocols for the limited extraction of lipids ranging from S1P to DAG, but excluding TAG, which is too hydrophobic to assess together with S1P. For instance, the simultaneous reversed phase LC analysis of all lipids within a sample, including highly hydrophobic substances, such as TAG, would be extremely time-consuming. Thus, we set up the first extraction with butanol, to recover the relatively hydrophilic lipids, and the second extraction with ethyl acetate/hexane, to recover the more hydrophobic lipids (termed BEH extraction) (Figure 1). Ethyl acetate/hexane (1:1) was suitable for extracting DAG, but not TAG (17:0/17:0/17:0), which was, instead, efficiently extracted with ethyl acetate/hexane (1:3). Extraction with chloroform/methanol (1:2) overnight at 48 °C (termed CM48 extraction) is widely used for sphingolipid analysis [18]. We compared the recovery of sphingolipids from samples extracted by the CM48 and BEH extraction methods. The BEH extraction was 80% more effective than the CM48 extraction, indicating that BEH extraction is sufficient for evaluating sphingolipids in small biological samples (*i.e*., less than 1 × 10^6^ cells) (Figure 2).

### 2.2. LC-MS/MS Method for Lipidomics

Although some isotope ions (M + 1) of PtdCho intrinsically produce MS/MS spectra identical to those of some SM molecular ions (M), each molecular species of PtdCho and SM could be separated under the reversed phase LC conditions. Just one set of chromatography conditions was sufficient for the detection of all target analytes. However, even after LC separation, the large amounts of PtdCho and PtdEtn components in the biological sample reduced the detection capability for acidic lipids, due to an ion suppression effect. To eliminate this undesired effect when acidic lipids were analyzed, a sample pretreatment procedure involving DEAE-cellulose column chromatography was introduced, to remove the neutral and basic lipids and thereby minimize ion suppression (Figure 1). The samples often appeared to be saturated with some molecular species of PtdCho, due to their abundance and high sensitivity of detection. Thus, the BEH extract prepared as described above was divided 1:9, and 10% of the BEH extract was used for the analysis of neutral and basic lipids, such as Sph, Cer, SM, PtdCho, PtdEtn and DAG. However, DAG was found to be heat-labile in the mass spectrometer ion source (200/300 °C (vaporizer/ion transfer temperature)), thus reducing the sensitivity of DAG detection. Therefore, lower-temperature MS conditions (200/250 °C (vaporizer/ion transfer temperature)) were required for the sensitive detection of DAG (Table 1). The remaining 90% of the BEH extract was fractionated by DEAE-cellulose chromatography for the analysis of acidic lipids, such as S1P, C1P, PtdSer, PtdOH and GM3. As in the case with DAG, lower-temperature MS conditions (100/200 °C (vaporizer/ion transfer temperature)) were required for the analysis of heat-labile lipids (PtdOH, PtdIns, PtdGro and GM3).

Our LC conditions included sequential prewashes of the injector needle with 10 mM phosphoric acid and methanol just prior to each sample injection, in order to suppress undesired interactions between the metal surfaces of the injection needle and S1P, C1P and PtdOH. This additive is needed for the high-sensitivity detection of acidic lipids [22]. However, the ability to detect DAG (14:0/14:0) was not proportional to its concentration around the detection limit, when the same injector-rinsing solvent was used. Therefore, for the analysis of DAG, phosphoric acid was not included in the rinsing solvent (Table 1).

**Table 1:** Summary of the SRM parameters and LC sample injector conditions.

Analyte	Precursor	Product	Collision (V)	Vaporizer/ion transfer temperature (°C)	MS method file	Prewash of sample injector
Sph	[M + H]^+^	236.3 (d16:1)	15	200/300	I	10 mM phosphoric acid followed by methanol
		238.3 (d16:0)				
		250.3 (d17:1)				
		264.3 (d18:1)				
		266.3 (d18:0)				
		292.3 (d20:1)				
Cer	[M-H_2_O + H]^+^	264.3 (d18:1)	20			
		266.3 (d18:0)				
HexCer	[M-H_2_O + H]^+^	264.3 (d18:1)	20			
		266.3 (d18:0)				
SM	[M + H]^+^	184.1	20			
PtdCho	[M + H]^+^	184.1	20			
PtdEtn	[M + H]^+^	[M + H − 141]^+^	20			
DAG	[M + NH_4_]^+^	[M + NH_4_ − 245.2]^+^ (14:0FA)	20	200/250	II	Water followed by methanol
		[M + NH_4_ − 271.3]^+^ (16:1FA)				
		[M + NH_4_ − 273.3]^+^ (16:0FA)				
		[M + NH_4_ − 297.3]^+^ (18:2FA)				
		[M + NH_4_ − 299.3]^+^ (18:1FA)				
		[M + NH_4_ − 301.3]^+^ (18:0FA)				
		[M + NH_4_ − 321.3]^+^ (20:4FA)				
		[M + NH_4_ − 345.3]^+^ (22:6FA)				
S1P	[M + H]^+^	236.3 (d16:1)	15	200/300	III	10 mM phosphoric acid followed by methanol
		238.3 (d16:0)				
		250.3 (d17:1)				
		264.3 (d18:1)				
		266.3 (d18:0)				
		292.3 (d20:1)				
		294.3 (d20:0)				
Cer1P	[M-H_2_O + H]	264.3 (d18:1)	20			
		266.3 (d18:0)				
PtdSer	[M + H]^+^	[M+H − 185]^+^	20			
PtdOH	[M + NH_4_]^+^	[M+NH_4_ − 115]^+^	20	100/200	IV	10 mM phosphoric acid followed by methanol
PtdGro	[M + NH_4_]^+^	[M+NH_4_ − 189]^+^	20			
PtdIns	[M + H]^+^	[M+H − 260]^+^	20			
GM3	[M+H]^+^	264.3 (d18:1)	50			
		266.3 (d18:0)				

### 2.3. Method Validation

In order to validate our approach developed in this study, we examined the recovery rate, precision, accuracy and linearity, with respect to lipid standards added to a suspension of 1 × 10^6^ WR/SMS1 cells. Commercially available synthetic lipid molecular species, which are difficult to detect in any biological sample, were used for this assay. Table 2 shows the recovery rate for each lipid standard. Table 3 and Supplementary Figure S1 show the precision, accuracy and linearity of the method. These validation data indicated that our method is suitable for the analysis of biological lipids in a complex biological matrix.

**Table 2:** Recovery rates of lipid standards added to the cell suspension. The values are expressed as the mean ± SD (*n* = 3).

Added lipid	Added amount	Recovery (%)	Extract
Sph (17:1)	50 pmol	114 ± 12	BEH extract
Cer (d18:1/12:0)	50 pmol	85 ± 5	
HexCer (d18:1/12:0)	50 pmol	91 ± 5	
SM (d18:1/12:0)	50 pmol	94 ± 1	
PtdCho (28:0)	500 pmol	117 ± 3	
PtdEtn (28:0)	500 pmol	85 ± 5	
DAG (14:0/14:0)	50 pmol	90 ± 8	
S1P (d17:1)	50 pmol	73 ± 1	Acidic fraction
Cer1P (d18:1/12:0)	50 pmol	83 ± 1	
PtdSer (28:0)	50 pmol	83 ± 2	
PtdOH (28:0)	50 pmol	105 ± 15	
PtdGro (28:0)	50 pmol	80 ± 15	
PtdIns (32:0)	50 pmol	75 ± 4	
GM3 (d18:1/12:0)	50 pmol	85 ± 17	

**Table 3:** Precision, accuracy and linearity of analyses of lipid standards added to the cell suspension.The linear regression equation was calculated from the log_e_-log_e_ plot of the MS peak area for each eluted lipid *versus* the amount of standard added. Data were obtained from three or four measurements. The values are expressed as the mean ± SD. RSD, relative standard deviation.

Added lipid	Amount (pmol)	Accuracy (%)	Precision (RSD%)	Detection limit (pmol)	Range (pmol)	Linear regression
Sph (17:0)	0.5	11.9	7.4	0.2	0.5–500	Y = 1.11X − 1.38 R2 = 0.999
	50	−3.1	9.4			
	500	5.2	8.6			
Cer (d18:1/12:0)	0.5	−19.3	10.8	0.05	0.5–500	Y = 1.11X − 2.86 R2 = 0.995
	50	−8.6	1.4			
	500	20.9	4.9			
GlcCer (d18:1/12:0)	1	20.5	9.7	0.2	1.0–500	Y = 0.981X − 1.62 R2 = 0.998
	50	8.5	7.5			
	500	−8.1	0.9			
SM (d18:1/12:0)	0.5	−6.9	15	<0.05	0.5–500	Y = 1.05X − 4.24 R2 = 0.999
	50	−1.8	1.3			
	500	8.9	4.7			
PtdCho (28:0)	5	1.6	5.8	<0.5	5–5,000	Y = 1.08X − 5.13 R2 = 0.995
	500	8.2	5			
	5,000	10.6	3.4			
PtdEtn (28:0)	0.5	−23.8	22.2	<0.5	0.5–5,000	Y = 0.933X − 1.561 R2 = 0.998
	500	−0.4	2.4			
	5,000	−6.9	4.6			
DAG (28:0)	0.05	−0.6	5.8	0.05	0.05–500	Y = 0.988X − 4.655 R2 = 0.999
	0.5	4.5	2.4			
	50	−2.3	5.6			
	500	6.3	4.8			
S1P (17:0)	0.5	4.1	4.7	0.05	0.5–500	Y = 0.995X − 3.817 R2 = 0.999
	50	−0.4	6.4			
	500	−4.9	1.6			
Cer1P (d18:1/12:0)	0.5	9.3	4.1	0.05	0.5–500	Y = 0.996X − 4.018 R2 = 0.997
	50	7.6	4.7			
	500	−15.8	3.1			
PtdSer (28:0)	0.5	−4.2	5.6	<0.5	0.5–2,500	Y = 1.024X − 3.126
	50	11.7	6.1			R2 = 0.999
	2,500	−14.9	1.4			
PtdGro (28:0)	1	−0.7	17.9	0.2	1.0–500	Y = 0.946X − 1.64 R2 = 0.999
	50	−1.8	14.7			
	500	1.3	4.4			
PtdIns (32:0)	1	−0.2	7.6	0.2	1.0–500	Y = 1.13X − 2.46 R2 = 0.999
	50	−14.0	4.3			
	500	10	3.3			
PtdOH (28:0)	1	5	5.8	0.2	1.0–500	Y = 1.26X − 2.66 R2 = 0.999
	50	−12.2	7.8			
	500	8.2	7.6			
GM3 (d18:1/12:0)	1	−8.3	8.3	0.2	1.0–500	Y = 0.951X − 0.663 R2 = 0.999
	50	18.1	6.7			
	500	−10.4	1			

### 2.4. Application to Biological Samples

**Figure 3:**
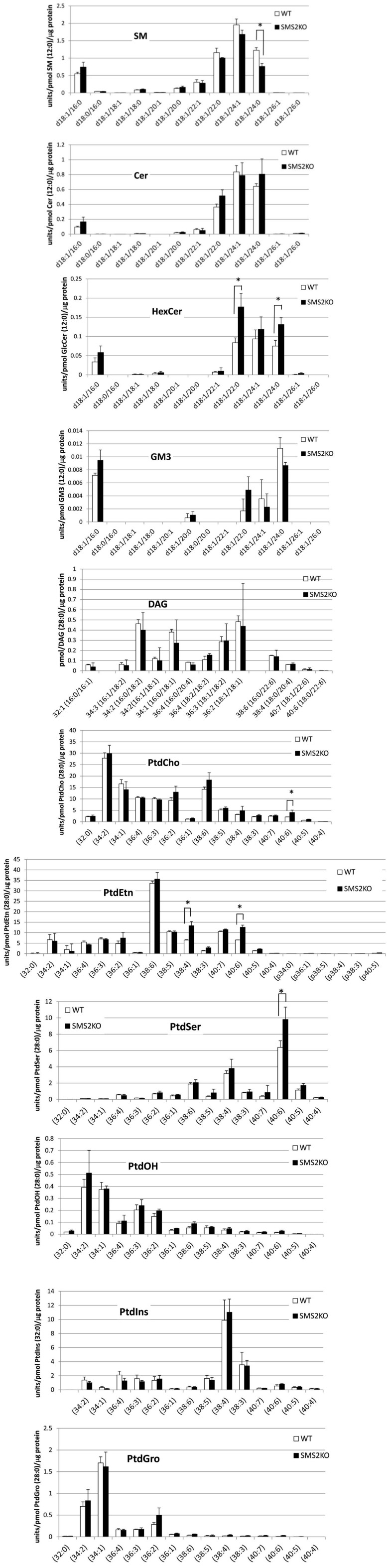
Differences in the levels of sphingolipid, GPL and DAG molecular species between wild-type (WT) and SMS2-KO mice. Three WT and three SMS2-KO mice were sacrificed, and their livers were excised and prepared for lipid analysis. The (34:2) contains the acyl structures of (16:0/18:2) and (16:1/18:1). The (36:3) predominantly contains the acyl structure of (18:1/18:2). The (36:4) and (38:4) predominantly contain (16:0/20:4) and (18:0/20:4), respectively. The (38:6), (40:6) and (40:7) predominantly contain the acyl structures of (16:0/22:6), (18:1/22:6) and (18:0/22:6), respectively. After standardization of the protein level, the ratio of the measured lipid’s peak area to that of the internal standard (1 pmol of each standard lipid) was represented as units/picomole internal standard/microgram protein. Data are expressed as the mean ± SD. * *p* < 0.05; Student’s *t*-test.

In this study, we developed a comprehensive analytical method for examining the link between sphingolipid metabolism and GPL metabolism via PtdCho and DAG. To demonstrate the application of the method to biological samples, we comparatively analyzed lipids derived from tissues of wild-type and SMS2-KO mice [26,27]. Significant differences in the levels of various lipid molecular species were found (Figure 3). For instance, the level of the d18:1/24:0 SM species was significantly lower in the liver tissue of SMS2-KO mice, as compared with the liver tissue of wild-type mice. The levels of the d18:1/22:0 and d18:1/24:0 species of HexCer were significantly higher in the livers of SMS2-KO mice. Intriguingly, the levels of docosahexaenoic acid (DHA, 22:6)-containing GPL species, *i.e*., PtdCho (40:6), PtdEtn (40:6) and PtdSer (40:6), were significantly higher in the livers of SMS2-KO mice. In addition, the level of PtdEtn (38:4), which is an arachidonic acid (20:4)-containing species, was also significantly higher in the livers of SMS2-KO mice. The changes in the levels of constituent lipids detected in the livers were not found in either the lungs or spleens of SMS2-KO mice. Therefore, our new method for analyzing the molecular species of sphingolipids and their surrounding lipids will facilitate future comprehensive analyses of membrane-associated and signal lipids.

## 3. Experimental Section

*Materials*: C17-sphingosine (Sph(d17:1)), C17-sphingosine-1-phosphate (S1P(d17:1)), 12:0-ceramide (Cer (d18:1/12:0)), 12:0-ceramide-1-phosphate (Cer1P (d18:1/12:0)), 12:0-glucosyl(β)ceramide (GlcCer (d18:1/12:0)), 12:0-sphingomyelin (SM (d18:1/12:0)), 14:0/14:0-diacylglycerol (DAG (14:0/14:0)), 14:0/14:0-phosphatidylcholine (PtdCho(28:0)), 14:0/14:0-phosphatidylserine (PtdSer (28:0)), 14:0/14:0-phosphatidylglycerol (PtdGro(28:0)) and 16:0/16:0-phopshatidylinositol (PtdIns (32:0)) were purchased from Avanti Polar Lipids (Alabaster, AL, USA). The 14:0/14:0-phosphatidylethanolamine (PtdEtn (28:0)) and triheptadecanoin (TAG (17:0/17:0/17:0)) were obtained from Wako Pure Chemicals (Osaka, Japan). Monosialoganglioside (GM3) was purchased from Nagara Sciences (Gifu, Japan). All solvents were HPLC or special grade, and other chemical reagents were analytical grade and were purchased from Nacalai Tesque (Kyoto, Japan). Ultrapure water was obtained from an RFU554CA ultrapure water system (Advantec, Tokyo, Japan).

*Lipid standard solutions*: Each lipid standard was dissolved in methanol or methanol/chloroform (2:1), to prepare solutions with concentrations ranging from 0.33–1.0 mg/mL. Each solution was diluted to 100 pmol/µL with methanol, except for PtdEtn and PtdSer, which were diluted with methanol/chloroform (2:1). These standard solutions were stored at −30 °C. Before use, lipids were diluted to the desired concentration with methanol and mixed to produce various standard cocktails. TAG (17:0/17:0/17:0) was dissolved in isopropanol.

*Biological samples*: WR19/Fas-SMS1 (abbreviated as WR/SMS1) cells were cultured in RPMI-1640 medium supplemented with 10% fetal calf serum, at 37 °C in a 5% CO_2_ atmosphere [24]. The resulting culture was used for the validation assay. SMS2-KO mice, generated in our laboratory, were maintained in a C57BL/6 background. Samples of various tissues collected from 24-week-old male mice were homogenized on ice with 900 µL of phosphate buffered saline (PBS) per 100 mg of wet weight, using a glass-Teflon homogenizer. A 10-µL aliquot of a 10% tissue homogenate suspension, equivalent to 1 mg wet weight of tissue, was used for lipid analyses. The concentration of protein in the tissue homogenate was determined using a BCA protein assay kit (Pierce, Rockford, IL, USA), after solubilization with 2% Triton X-100. Experiments were performed under the guidelines of the animal ethics committee of Kanazawa Medical University.

*Sample preparation*: Cultured cells (1 × 10^6^ cells/50 µL in PBS) or homogenized tissue (1 mg wet weight/50 µL in PBS) were prepared in 1.5 mL polypropylene vials, and 50 pmol of each commercially available standard lipid (Sph (d17:1), S1P (d17:1), Cer (d18:1/12:0), Cer1P (d18:1/12:0), GlcCer (d18:1/12:0), SM (d18:1/12:0), DAG (14:0/14:0), PtdCho (28:0), PtdSer (28:0), PtdGro (28:0), PtdIns (32:0) and PtdEtn (28:0)) and 500 pmol of PtdCho and PtdEtn, in 50 µL of methanol, were added as an internal standard. The MS peak areas of the measured lipids were compared with those of the internal standards. On the other hand, in order to obtain validation data, the desired concentration of each lipid standard was added to a suspension of WR/SMS1 cells (1 × 10^6^ cells/50 µL in PBS) and then analyzed without the use of any internal standards in MS. Validation parameters were evaluated using absolute peak areas.

Next, 30 µL of 500 mM phosphate buffer (pH5.8), 170 µL of water and 750 µL of butanol were added to each sample on ice. Subsequent procedures were performed at room temperature. After vigorous shaking for 5 min and sonication for 3 min in an ultrasonic bath (Branson 5510, Branson, Soest, The Netherland), the samples were centrifuged at 1,000 × *g* for 5 min, after which, 700 µL of the upper layer was collected in a 2-mL polypropylene vial. The original suspension was re-extracted by the addition of 350 µL of ethyl acetate and 350 µL of hexane, followed by centrifugation. The resulting extract was combined with the first butanol extract. After the addition of 700 µL of methanol, this extract was then diluted 1:9 (*i.e*., 210 and 1,890 µL). A volume equivalent to 10% of the extract was dried under diminished pressure at 30 °C (Taitec VC-36S, Taitec, Saitama, Japan) and then re-dissolved in 60 µL of LC mobile phase B (described below) and 90 µL of mobile phase A, added sequentially. This sample was used for the analysis of Sph, Cer, HexCer, SM, PtdCho and PtdEtn. The remaining 90% of the extract was fractionated on a DEAE-cellulose (Wako Pure Chemicals) column (500 µL bed volume packed in a 1,000 µL polypropylene pipet tip). After washing with 2,000 µL of methanol, the column-bound lipids were eluted with 1,000 µL of methanol/28% aqueous ammonia/formic acid (1,000:33:22). The organic solvent was evaporated from the eluate under diminished pressure at 30 °C, after which, mobile phase A was added to a final volume of 150 µL (Figure 1). The resulting sample was used for the analysis of acidic lipids (*i.e*., S1P, Cer1P, GM3, PtdSer, PtdGro, PtdIns and PtdOH).

*LC-MS/MS*: The Ultimate 3000 LC system (Thermo-Fisher Scientific, Waltham, MA, USA) used in the study was controlled with Chromeleon software. The autosampler was equipped with a non-metallic 6-port valve, and the injection program was set up to wash the metal injector needle with 10 mM phosphoric acid and methanol in sequence, just prior to each sample injection. For analyses of DAG only, water was used for the needle prewash, instead of 10 mM phosphoric acid (Table 1). Chromatography was performed as described previously [22], with several modifications. Briefly, lipids were separated on an Acclaim PepMap100 C18 (3 µm, 150 × 1.0 mm i.d.) column (Thermo-Fisher Scientific). Mobile phase A was isopropanol/methanol/water (5:1:4) with 0.2% formic acid, 0.028% ammonia and 5 µM phosphoric acid. Mobile phase B was isopropanol with 0.2% formic acid and 0.028% ammonia. The gradient conditions were as follows: 30% B to 50% B over 2 min, to 80% B over 10 min, to 95% B over 0.5 min and holding at 95% B for 3.5 min, then from 95% B to 5% B over 0.5 min and holding at 5% B for 1.5 min and, finally, to 30% B over 4 min. The flow rate was 45 µL/min, and the chromatography was performed at 40 °C. Typically, 5 µL of sample were injected.

**Figure 4:**
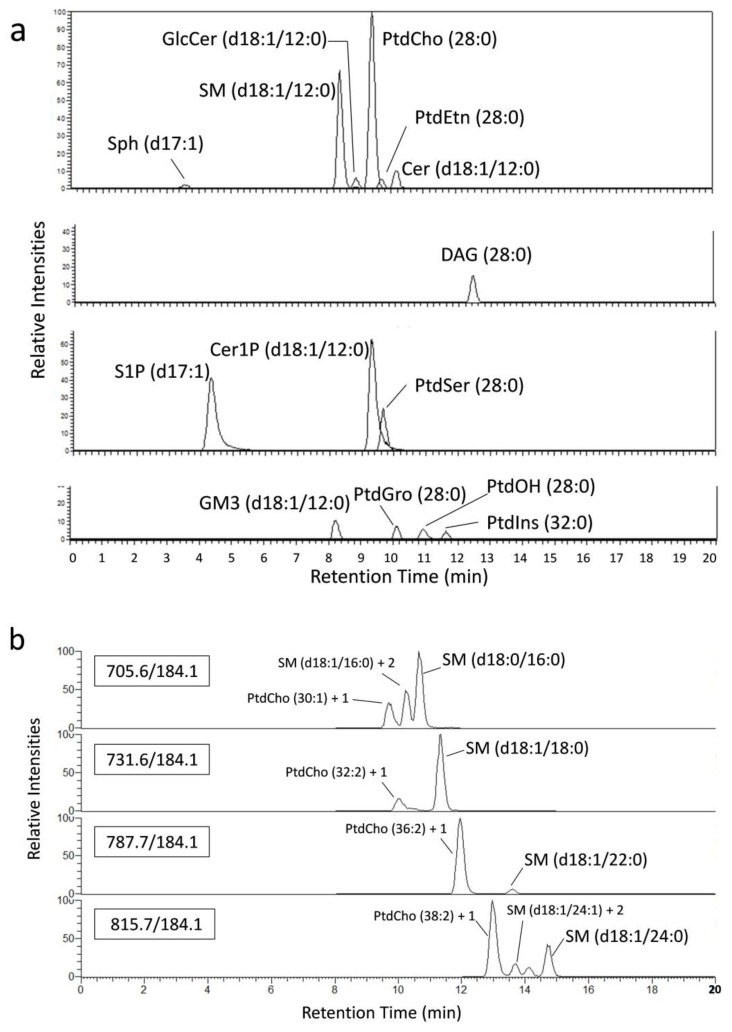
Mass chromatograms of the standard lipids added to the cell suspension. (**a**) 50 pmol of each synthetic standard was added to a cell suspension (1 × 10^6^ cells), and the standard lipids were detected by the present method. Peak intensities are represented as relative values to PtdCho (28:0). (**b**) Each biological SM molecular species was detected without overlapping with the naturally occurring 13C isotopic signals of the other PtdCho and/or SM molecular species on the chromatograms. The 13C isotope ions [M + 1]^+^ of PtdCho (30:1) and [M + 2]^+^ of SM (d18:1/16:0) are abbreviated as “PtdCho (30:1) + 1” and “SM (d18:1/16:0) + 2”, respectively.

The LC system was coupled on-line to a TSQ Vantage mass spectrometer (Thermo-Fisher Scientific) equipped with an electrospray ionization (HESI II probe) source. The ion spray voltage was set to 3.0 kV in the positive ion mode, and the heated capillary and vaporizer temperatures were varied depending on the lipid class, as shown in Table 1. Analytes were detected in the selected reaction monitoring (SRM) mode. The mass transitions and parameters are shown in Table 1. The Q1 and Q3 peak widths were set to 0.4 and 0.7, respectively. Other parameters were set according to the manufacturer’s recommendations. The MS system was controlled using the Xcalibur software. Each molecular species was identified based on the MS/MS spectrum and the LC retention time (Figure 4).

## 4. Concluding Remarks

Chloroform and methyl-*t*-butyl ether, which are often utilized for lipid extraction, were not employed in our methods, to allow the use of polypropylene labware throughout the sample preparation process [28]. This advantage makes our method suitable for automated applications in clinical studies [29] and for pathologic and diagnostic studies of sphingolipid metabolism. The present method is for comparative analyses, rather than for quantitation, because it does not show the response factor for each biological lipid target of interest in comparison to the internal standards.

## Supplementary Materials

Supplementary materials can be accessed at: http://www.mdpi.com/2218-1989/4/1/98/s001.

## Supplementary Files

Supplementary File 1 Supplementary Materials (PDF, 427 KB)
