# Supplementary material for: Comparative Analysis of Biological Sphingolipids with Glycerophospholipids and Diacylglycerol by LC-MS/MS
Source: Metabolites. 2014 Jan 27;4(1):98–114. doi: 10.3390/metabo4010098 (PMC4018675; doi:10.3390/metabo4010098)

Supplementary Materials

Figure S1. Linearity of each standard lipid used to spike the cell suspension. A suspension containing 1 × 10^6 WR/SMS1 cells was spiked with lipid standards and analyzed by the present method, without the use of any internal standards during MS. Data are presented as the $\log_e$-$\log_e$ plot of the MS peak area for each eluted lipid *versus* the amount of standard added.

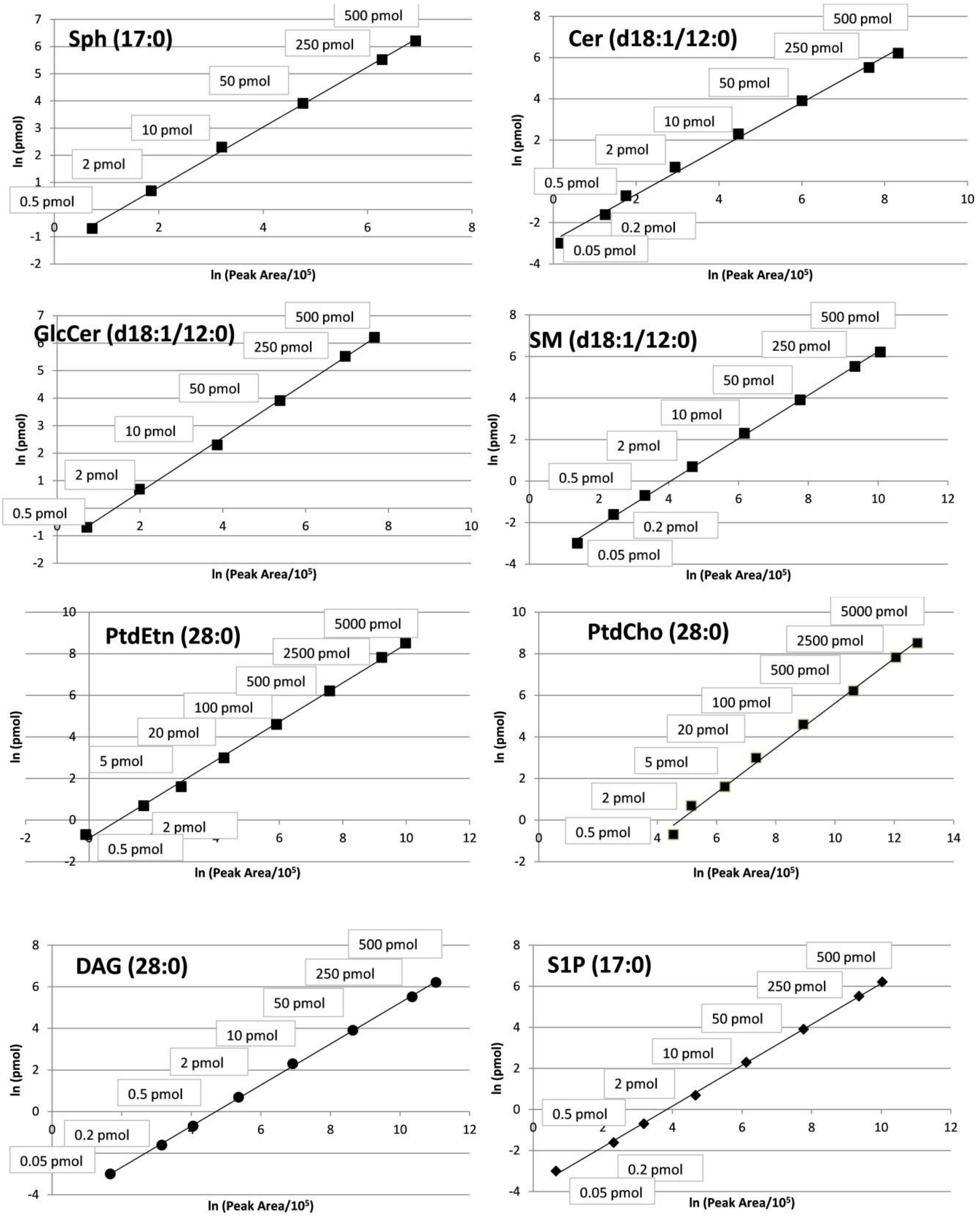

Figure S1. *Cont.*

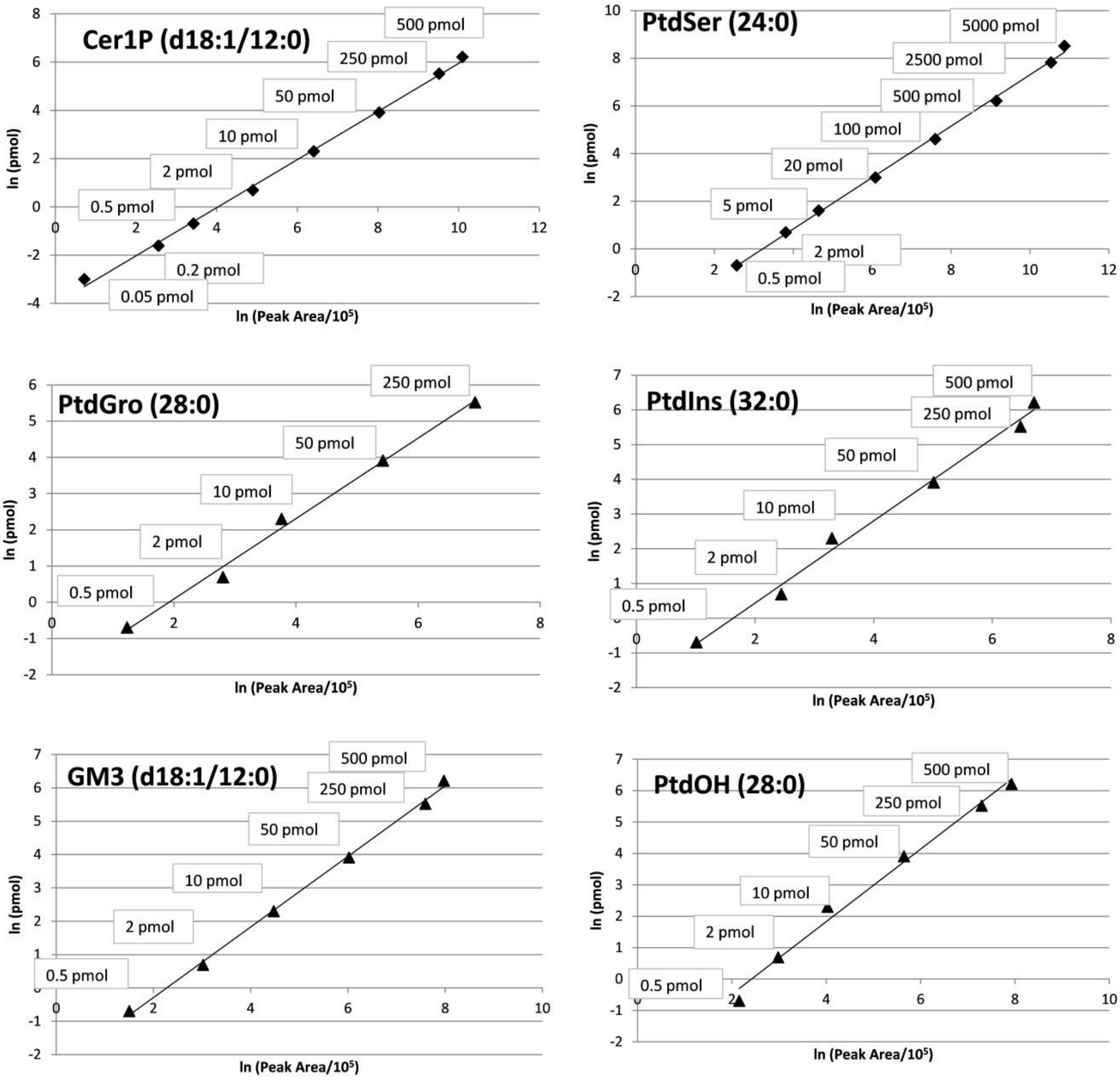

Supplement: Supplementary File 1 — Supplementary Materials (PDF, 427 KB) [file metabolites-04-00098-s001.pdf]
